# A standard cytogenetic map of *Culex quinquefasciatus* polytene chromosomes in application for fine-scale physical mapping

**DOI:** 10.1186/s13071-015-0912-4

**Published:** 2015-06-06

**Authors:** Maria F Unger, Maria V Sharakhova, Adam J Harshbarger, Patrick Glass, Frank H Collins

**Affiliations:** Department of Biological Sciences, University of Notre Dame, Notre Dame, IN USA; Department of Entomology, Virginia Tech, Blacksburg, VA USA; Laboratory of Evolutionary Cytogenetics, Tomsk State University, 36 Lenina Avenue, Tomsk, 634050 Russia

**Keywords:** Physical mapping, Culex fatigans, Culex pipiens, Culex quinquefasciatus, Polytene chromosomes, Gene mapping, Chromosomal map, FISH, Assembly, Misassembly, Cytogenetic map

## Abstract

**Background:**

Southern house mosquito *Culex quinquefasciatus* belongs to the *C. pipiens* cryptic species complex, with global distribution and unclear taxonomy. Mosquitoes of the complex can transmit human and animal pathogens, such as filarial worm, West Nile virus and avian malarial *Plasmodium.* Physical gene mapping is crucial to understanding genome organization, function, and systematic relationships of cryptic species, and is a basis for developing new vector control strategies. However, physical mapping was not established previously for *Culex* due to the lack of well-structured polytene chromosomes.

**Methods:**

Inbreeding was used to diminish inversion polymorphism and asynapsis of chromosomal homologs. Identification of larvae of the same developmental stage using the shape of imaginal discs allowed achievement of uniformity in chromosomal banding pattern. This together with high-resolution phase-contrast photography enabled the development of a cytogenetic map. Fluorescent in situ hybridization was used for gene mapping.

**Results:**

A detailed cytogenetic map of C. quinquefasciatus polytene chromosomes was produced. Landmarks for chromosome recognition and cytological boundaries for two inversions were identified. Locations of 23 genes belonging to 16 genomic supercontigs, and 2 cDNA were established. Six supercontigs were oriented and one was found putatively misassembled. The cytogenetic map was linked to the previously developed genetic linkage groups by corresponding positions of 2 genetic markers and 10 supercontigs carrying genetic markers. Polytene chromosomes were numbered according to the genetic linkage groups.

**Conclusions:**

This study developed a new standard cytogenetic photomap of the polytene chromosomes for *C. quinquefasciatus* and was applied for the fine-scale physical mapping. It allowed us to infer chromosomal position of 1333 of annotated genes belonging to 16 genomic supercontigs and find orientation of 6 of these supercontigs; the new cytogenetic and previously developed genetic linkage maps were integrated based on 12 matches. The map will further assist in finding chromosomal position of the medically important and other genes, contributing into improvement of the genome assembly. Better assembled *C. quinquefasciatus* genome can serve as a reference for studying other vector species of *C. pipiens* complex and will help to resolve their taxonomic relationships. This, in turn, will contribute into future development of vector and disease control strategies.

**Electronic supplementary material:**

The online version of this article (doi:10.1186/s13071-015-0912-4) contains supplementary material, which is available to authorized users.

## Background

The southern house mosquito *Culex quinquefasciatus* Say 1823 is a primary vector of several human and animal pathogens: A) lymphatic filarial worm *Wuchereria bancrofti*, which causes elephantiasis of limbs and genitalia, causing life-long disabilities; B) West Nile virus and several other encephalitis-causing arboviruses which can be deadly; C) Avian malarial *Plasmodium*, which makes *Culex* a good model for studying malaria transmission. The burden of these diseases is enormous, with millions of people affected, and even more at risk. Since there is no human vaccine available for the listed viral diseases, and drugs for lymphatic filariasis can treat the worms but cannot alleviate the developed symptoms such as lymphedema, elephantiasis and hydrocele, vector control is the most efficient method for disease prevention, according to the Center for Disease Control and World Health Organization. Additionally, *C. quinquefasciatus* is a member of a cryptic species *Culex pipiens* complex [1] which has near-worldwide distribution. The complex includes at least three [2] or more [3, 4] mosquito species, and, two eco-forms or subspecies, which are morphologically identical, except for the structure of male genitalia. Nevertheless, these species differentiate by blood-meal host preference, ability to diapause, being autogenous or non-autogenous, by larval ecology and some other physiological characteristics. The systematic relationships of *C. pipiens* complex species are still under debate, and the vectorial capacity of each species is under investigation [4]. Speciation, in general terms, occurs not only by gene mutations, but also to chromosomal rearrangements, especially inversions. Thus, finding the location of genes in a genome helps to improve our understanding of genome organization, contributes towards deciphering how the genome functions, and provides possible landmarks of genome organization like inversions that may be associated with different taxa in the complex. Studying the *C. quinquefasciatus* genome organization is the first step towards clarification of *C. pipiens* complex relationships. Additionally, as a required framework against which phenotypes and genotypes can be associated in population studies, it can form an improved basis for the development of new vector control and disease prevention strategies.

The South African Johannesburg (JHB) strain of *C. quinquefasciatus* was sequenced independently by two sequencing centers: Broad Institute and J. Craig Venter Institute (formerly known as TIGR) in 2007 [5]. The merged consensus assembly of TIGR and Broad independent assemblies resulted in a 579 Mbp genome assembly consisting of 3,171 supercontigs. About 10 % of these supercontigs of various sizes were assigned to genetic linkage groups because sequenced genetic markers could be co-located to specific supercontigs [6]. However, genetic linkage mapping reveals only the relative positions of these supercontigs in each of three linkage groups. Recently reported mitotic chromosomes based physical mapping [7] reveals physical location of 37 supercontigs, but this method lacks resolution, reporting, for example two supercontigs 3.205 and 3.99 belonging to the same location. Also, due to the short length of mitotic chromosomes it is impossible to infer the supercontig orientation within the chromosome, which is important for advancing genome assembly. Hence, the high-resolution chromosomal locations of the 3,171 genomic supercontigs remain unknown. Thus, polytene chromosome based physical mapping was undertaken to improve the quality of the current genome assembly of *C. quinquefasciatus.*

The development of a physical map for the members from *C. pipiens* complex represents a great challenge because of a number of reasons. 1) *C. pipiens* and *C. quinquefasciatus* develop only a few nuclei with readable polytene chromosomes per salivary gland [8]; 2) telomeres of *Culex* polytene chromosomes often connect to each other [8, 9], making it difficult to discern where one chromosomal arm ends and another begins; 3) non-sister chromatids form ectopic contacts throughout their length, and these fusions impede a good spreading of the chromosomes on the slide.

The first reported photograph of polytene chromosomes was based on a US strain of *C. pipiens* reported by Kitzmiller and Clark in 1952 [10]. This was followed by Dennhöfer in 1968 who analyzed an autogenous *C. pipiens “molestus”* colony from Germany. She later revised and corrected [11] her initial drawn map [9]. However, Dennhöfer’s parallel attempts to produce a map for a non-autogenous *C. pipiens fatigans*, which is a synonym for *C. quinquefasciatus* [2], were not successful, because the chromosomes were very difficult to spread on the slide [9]. Dennhöfer’s initial work was followed by the drawn map of a strain of *C. fatigans* from India [12]. Finally, in attempts to unify all previous maps, a drawn map of *C. pipiens* from Japan was published by Tewfik and Barr in 1974 [13]. At that time the first paracentric inversion on the 2R chromosome in *C. quinquefascuatus* was also described [14]. However, none of these drawn maps corresponded consistently with each other, probably because the polytene nuclei were derived from different tissues, from comparable tissues dissected at different development times, and possibly because different members of the species complex were being analyzed.

The first photographic map was constructed for *C. pipiens* in 1998 by Zambetaki *et al.* [15]. It featured photographs of lacto-aceto-orcein stained chromosomes from adult Malpighian tubules, but lacked the details necessary for physical mapping. The introduction of West Nile Virus into the US in 1999 followed by its spread into Central and South America stimulated cytogenetic research for *C. pipiens* complex species and resulted in the development of additional photographic maps. The work by Campos with coauthors [16], describes a method of obtaining stained chromosomes from pupal and adult Malpighian tubules for a Brazilian strain of *C. quinquefasciatus*. A photo map of salivary gland chromosomes from 4th instar larvae of the JHB strain of *C. quinquefasciatus* was published in 2007 by McAbee *et al.* [8]. Unlike the previous studies, this map was based on unstained chromosomes photographed under phase-contrast microscope using digital technology. This study developed some chromosome landmarks and described inversions on both arms of one of the autosomes. However, this publication reported 2 to 3 variants of each chromosomal arm, and the banding pattern was not consistent across homologous arms, probably due to the subtle differences in the developmental stages of larvae used for chromosomal preparation. It also was not integrated with the genetic linkage map of *C. quinquefasciatus* that was available at the time [17].

Here we present a new cytogenetic map of the *C. quinquefasciatus* JHB strain, based on polytene chromosomes from salivary glands of IVth instar larvae. Our map was developed by utilizing high resolution photographs of chromosomes with a high degree of polytenization with reproducible banding patterns. Chromosome images were completely straightened and robust landmarks to distinguish chromosome arms were established. This map adopted a chromosome nomenclature of the genetic linkage map developed for the *C. pipiens* complex [6] and for mitotic chromosomes of *Culex quinquefasciatus* [7]. The polytene and linkage maps were matched by placement of two genetic markers and 10 supercontigs carrying genetic markers to the chromosomes, based on fluorescent *in situ* hybridization (FISH) results. A total of 16 genomic supercontigs were mapped. The orientation of six of these supercontigs was inferred and one was considered to be putatively misassembled.

## Methods

### Raising mosquito larva for chromosome preparations

To ensure high quality polytene chromosome preparations, mosquito larvae of the Johannesburg strain of *C. quinquefasciatus* were raised at low density 15–20 larvae per 2 L of distilled water. Every other day, 2 ml of food (2.5 g baker’s yeast, 2.5 g of Bovine Liver powder mixed in with 250 ml of distilled water) was added to the larval pans. Larvae were raised on 14 °C with a 18 h light/6 h dark cycle.

The inbreeding stock strains were raised at RT and natural lighting throughout the year. One or two egg rafts of each generation were raised at lower temperature with the conditions described above, and used for the chromosome preparations.

### Chromosome preparation

Preparations of polytene chromosomes were made from fixed mosquito larvae, as described in [8], with the exception of the dissection method and the sub-staging within IV instar larvae. Freshly fixed larvae (minimum 48 h after fixation until 3–4 days) were found best for slide-making, as older fixations tend to become “stiff” and unusable for slide-making. Only larvae with slightly oval IDs were selected for the dissection. If the larvae featured large discs which had obvious leg-shape, such larvae was discarded. This oval shape of IDs was identified for our inbred strains, and possibly would not be the same for other colonies. For other *Culex* strains the precise shape of IDs would have to be confirmed for each individual colony, and could vary between mosquito colonies. We used thin needles throughout our dissections. The glands were dissected at their top part where they were attached to the elementary canal, and placed to the slides (one gland per slide) in a small drop of 50 % propionic acid. Each gland was macerated and covered with a cover slip. After that, the cover slip was moved one-two times side-ways, without direct pressure. An additional small amount of propionic acid was added to ensure the saturation of the gland. After about 5–10 min the slide was covered by filter paper and the gland was squashed using extremely gentle tapping, as *Culex quinquefasciatus* chromosomes are prone to breakage. After that, the chromosome preparation was examined under the microscope and additionally squashed if needed. Then, the slide was placed on the warmer (37 °C) for approximately one minute, to adhere chromosomes to the slide. Next, the slides were kept for a couple of hours in the cool dark humid chambers to collect enough slides for the next step, after which they were dipped in liquid nitrogen. Cover slips were removed with a razor blade, and slides were dehydrated in a series of ethanol washes (50 %, 70 %, 90 %, 100 %). After that, the quality of these slides was examined under the phase-contrast microscope.

### Chromosome map development

Chromosome preparations of a supreme quality were photographed under the 100X objective, covered with a cover-glass using 100 % ethanol as a media. An Olympus BX60 microscope and an Olympus DP-72 camera (Olympus Corporation of the Americas) were used to obtain the photographs. Images of the best chromosomes were stitched together manually using Adobe Photoshop software (Adobe Software, Mountain View, CA). After that, chromosome arms were straightened using Adobe Photoshop. At least five images of each arm were used in order to overlay them and achieve an accurate representation of the most common banding pattern.

### *In situ* hybridization: probe preparation

Genomic DNA was extracted from *C. quinquefasciatus* pupae using a DNeasy Blood & Tissue Kit (Qiagen, Hilden, Germany), so as to avoid bacterial contamination of the DNA. The 15 largest supercontigs were then chosen from the genome using a custom Perl script, which sorts the genomic supercontigs of the CpipJ1 assembly by length. For each of the chosen 15 supercontigs, two unique exons were found in close proximity to the 3′ and 5′ ends, based on the assembly data from VectorBase [18]. Primers for each of these exons were designed using GeneFisher2 primer design software [19], and checked using OligoAnalyzer 3.1 [20]. The PCR conditions were as follows: 95 °C for 5 min; 45 cycles of (95 °C for 30s, 56 °C for 30s, 72 °C for 45s); 72 °C for 5 min; 4 °C indefinitely. The annealing temperature was sometimes changed based on varying primer melting temperatures. The products were then size-separated in 0.5 % agarose gel. After visualization on the UV-table, the bands of expected size were excised from the gel and purified using an Illustra GFX PCR DNA and Gel Band Purification Kit (GE Healthcare, Buckinghamshire, UK).

### *In situ* hybridization: probe labeling

A Random Primer labeling Kit (RPLK): (Invitrogen, catalog number: 18187–013) was used to label each probe with Cy3-dUTP (GE Healthcare, catalog number: PA53022) for the 5′-flank of a supercontig probe, or with Cy5-dUTP (GE Healthcare, catalog number: PA55022) for the 3′-flank of a supercontig probe (GE Healthcare PA53022 and PA55022 respectively). From 500 to 1000 ng of probe DNA was labeled as proposed by the RPLK protocol. Each probe was resuspended in 10 μl of 2x Hybridization buffer, with the buffer recipe based on that described in [21]. Two probes of distinct colors, each corresponding to one of the supercontig’s flanks, were mixed and hybridized simultaneously to polytene chromosomes. To simplify mapping, chromosome images were taken before and after FISH.

### Fluorescent *in situ* hybridization (FISH)

FISH was conducted exactly as described earlier [22], with the exception that the hybridization temperature overnight was 42 °C. The fluorescent signals were detected and recorded using a GE Healthcare DeltaVision deconvolution microscope and a Nikon A1-R confocal microscope, using the Imaging Facility Core (NDIIF).

## Results

### A standard photographic map of *C. quinquefasciatus* JHB polytene chromosomes

**Fig. 1:**
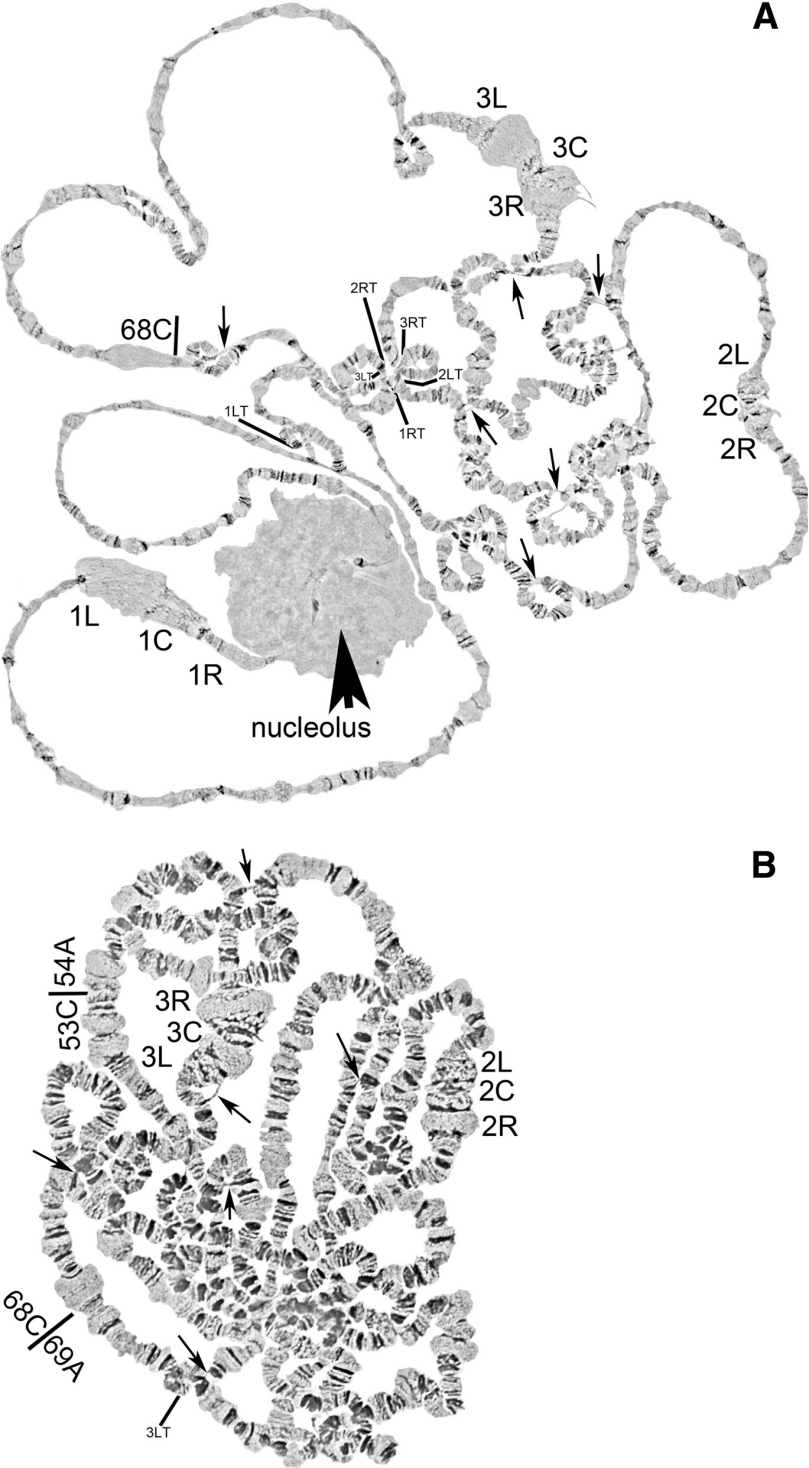
Typical chromosomal layouts from salivary glands of IV instar larvae *C. quinquefasciatus*, (**a**) Generation four of an iso-female JHB line, and (**b**) Generation seven of iso-female JHB line. Centromeres and chromosome arms are labeled. Thin arrows point to the ectopic contacts. Nucleolus is shown by thick arrow (**a**). Landmark of 3L is shown in subdivision 68C (**a**, **b** ). Telomeres are abbreviated as T, and shown where possible

**Fig. 2:**
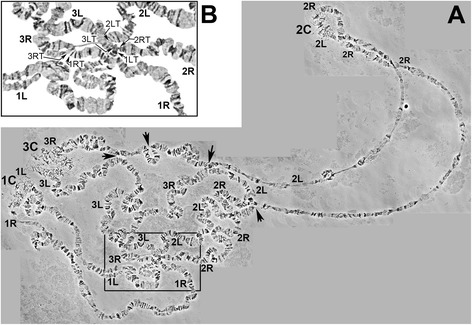
A typical layout of *C. quinquefasciatus* chromosomes, (**a**), and a zoom-in of the telomeric fusion area in (**b**). Ectopic contacts are pointed out by thick arrows. Telomeric regions are pointed out by thin arrows. Telomeres are abbreviated as T, right arms as R, left arms as L, centromeres as C

In order to develop a cytogenetic map for the polytene chromosomes of *C. quinquefasciatus* we examined salivary glands, Malpighian tubules, and gastric cecum of 4^th^ instar larvae, pupae and adults, for the presence of polytene chromosomes using lacto-aceto-orcein staining. All of these tissues develop many lower-polytenized nuclei but only a few are highly polytenized. We chose to work with salivary glands, because they appeared to have chromosomes which achieved the highest polyteny level and their banding patterns did not vary as much as in chromosomes of other tissues. We also utilized Imaginal Disks (IDs) as a pointer to the sub-staging within IV instar larval stage. Larvae possessing IDs with oval shape were found to be the best candidates for well-developed polytene chromosomes, with a more uniform banding pattern. In contrast, larvae with IDs round (earlier sub-stage), or with IDs leg-shaped (later sub-stage), had respectively under-developed or over-developed polytene chromosomes, respectively. For the map development we used iso-female inbred lines of JHB strain to decrease inversion polymorphism and asynapsis of homologs. The best chromosome photographs were obtained from iso-female lines of the JHB strain generation 4 (Fig. 1a) and generation 7 (Fig. 1b, Fig. 2). Approximately 25 % of all chromosome preparations contained more than one nucleus suitable for cytogenetic analysis. Images were taken from approximately 30 of the best slides. Chromosome arms were straightened and overlaid using AdobePhotoshop. Images were compared to identify a common banding pattern for the *C. quinquefasciatus* chromosomes and establish landmarks for chromosome arm recognition.

**Fig. 3:**
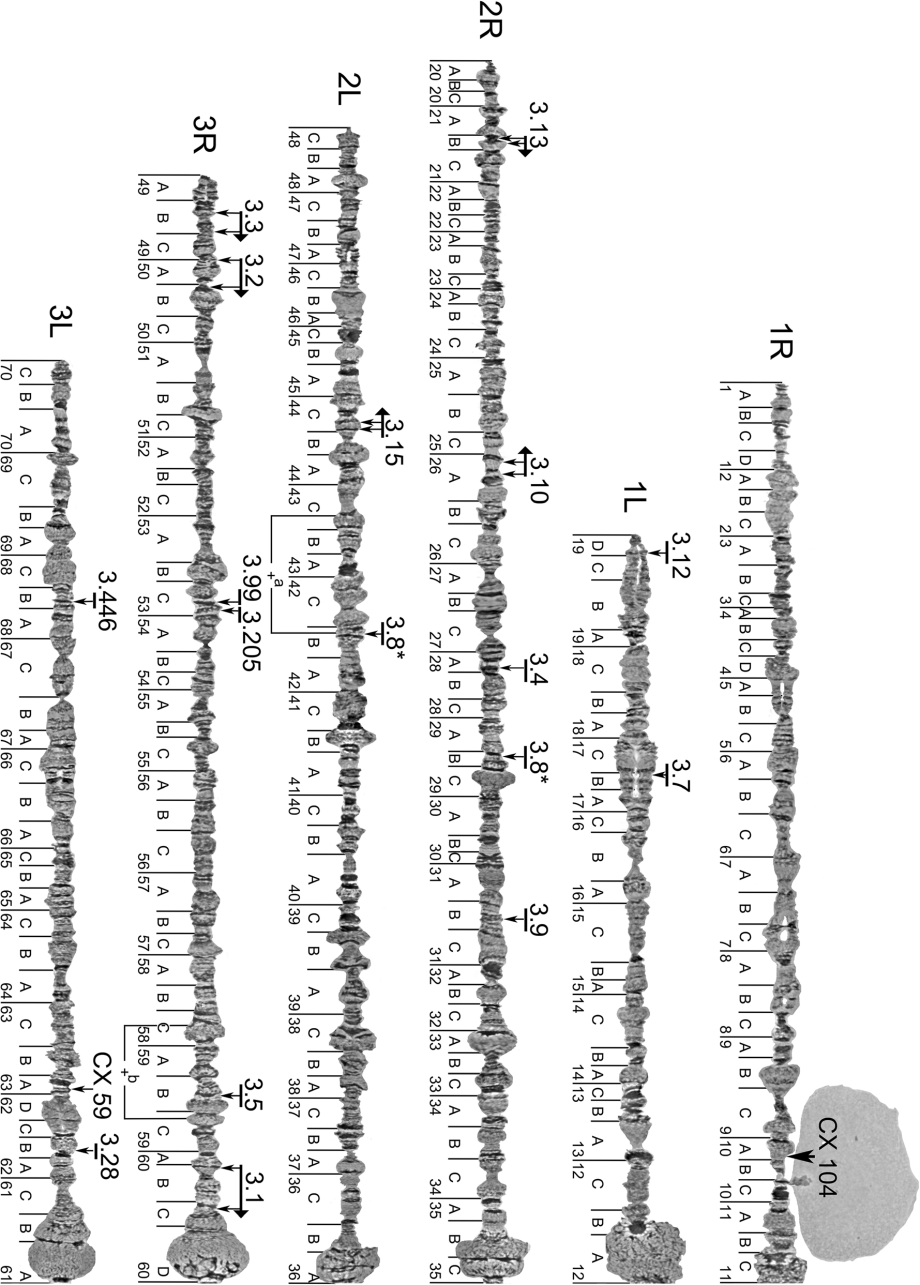
A photomap of polytene chromosomes from *C. quinquefasciatus* salivary glands and results of physical mapping. Chromosomal arms are abbreviated as R for right arms, and L for left arms. Polymorphic inversions are shown below the chromosomes. Locations of the FISH signals on the chromosomes are shown with the thin arrows, above the chromosomes. The corresponding signals to the supercontigs are shown above those thin arrows as horizontal thick arrows or as horizontal lines, if the orientation of supercontig is unknown. The names of the supercontigs are shown with the numbers above the large arrows and lines. In case if a genetic marker did not have a correspondence to any supercontig, only the genetic marker name is shown. A supercontig with ambiguous location is marked with an asterisk*. Please see Table 1 for further information about hybridized DNA probes which correspond to supercontigs and genetic markers

*C. quinquefasciatus* has a karyotype of 2n equal to 6. The cytogenetic map includes 3 chromosomes numbered from 1 to 3 according to the genetic nomenclature developed for *C. quinquefasciatus* [6]. The correspondence between chromosomes and genetic linkage groups was determined through the hybridization of six genetic markers to the chromosomes, using fluorescently labeled cDNA. An additional physical mapping of eleven unique sequences from eight supercontigs, also carrying genetic markers, contributed to further correspondence between the cytogenetic and genetic maps. A total of 14 matches were established based on the results of FISH (Fig. 3, Table 1). The smallest sex-determining chromosome [23] and two autosomes were numbered as 1, 2 and 3 respectively based on prior assignment as numbered linkage groups. Each chromosome of *C. quinquefasciatus* in polytene complement has two arms. The longer arms were designated as the right (R) and shorter arm as the left (L) arm with the most dense pericentromeric band considered as the centromere and used as an arm divider. The map divisions were created following the method of Bridges [24], where approximately each subdivision starts with a strong and easily recognizable band and includes ~5 bands. For each chromosome, the numbering was contiguous through the centromeres, starting from the telomere of an arm R to the telomere of an arm L. Even though after completion of the map we found that it looks most similar to one of the drawn maps [12], it is important to emphasize that subdivisions on our map have no correspondence to previous cytogenetic map divisions.

**Table 1:** A list of physically mapped genomic supercontigs and cDNAs of *C. quinquefasciatus*

Supercontig name or (Genetic marker)	Corresponding gene name or GB accession number	Gene start location in supercontig	Chromosome arm (subdivision) by physical mapping	Direction left to right	Chromosome distance (in centi-Morgans) by genetic linkage map [6]
(CX104)	FD664721	n/a	1R (10A)	n/a	1 (10.3 cM)
3.12	CPIJ001096	1,880,889	1L (19D)	n/a	1 (29.5 cM)
3.7	CPIJ000579	n/a	1L (15B)	n/a	not mapped
3.13	CPIJ001097	39,763	2R (21B)	Forward	2 (6.2 cM)
3.13	CPIJ001130	1,013,903	2R (21B)		not mapped
3.10	CPIJ000758	132,168	2R (26A)	Reverse	not mapped
3.10	CPIJ000786	1,479,122	2R (26A)		not mapped
3.4	CPIJ000419	1,663,135	2R (28A)	n/a	2 (25.3 cM)
3.8*	CPIJ000842	1,844,409	2R (29B)	n/a	not mapped
3.9	CPIJ000932	1,994,460	2R (31B)	n/a	not mapped
3.8*	CPIJ000807	367,208	2L (42B)	n/a	not mapped
3.15	CPIJ001243	224,054	2L (44C)	Forward	not mapped
3.15	CPIJ001288	956,670	2L (44C)		not mapped
3.3	CPIJ000183	1,517,868	3R (49B)	Forward	3 (1.9 cM)
3.3	CPIJ000244	2,734,241	3R (49B)		3 (1.9 cM)
3.2	CPIJ000248	128,985	3R (50A)	Forward	3 (5.1 cM)
3.2	CPIJ000339	2,703,669	3R (50B)		3 (5.1 cM)
3.99 (CX112)	FD664727	305,099	3R(53C)	n/a	3 (22.0 cM)
3.205 (CX17)	FD664699	96,983	3R (53C)	n/a	3 (22.3 cM)
3.5	CPIJ000458	452,949	3R (59B)	n/a	3 (39.6 cM)
3.1	CPIJ000004	373,018	3R (60B)	Forward	3 (40.0 cM)
3.1	CPIJ000147	3,834,983	3R (60C)		3 (40.0 cM)
3.28 (CX51)	GT056146	430,035	3L(62B)	n/a	3 (41.6 cM)
(CX59)	FD664716	n/a	3L (63A)	n/a	3 (49.5 cM)
3.446 (CX11)	FD664697	316,789	3L(68B)	n/a	3 (55.6 cM)

A supercontig with ambiguous location is marked with an asterisk*

As it was noted previously [9] chromosomes of *C. quinquefasciatus* do not form a distinct chromocenter. Pericentromeric regions are usually located on the periphery of the nucleus and can be easily identified by their puffy, mesh-like structure (Figs. 1 and 2). The pericentromeric regions of *C. quinquefasciatus* chromosomes have reproducible morphology and can be considered as major landmarks for chromosome recognition: subdivisions 11C-12A for chromosome 1; 35B–36B for chromosome 2; 60C–61B for chromosome 3 (Figs. 1, 2 and 3). Interestingly, telomeric regions of *C. quinquefasciatus* are often connected to each other (Fig. 2), as it was described for *C. pipiens* [9]. This feature makes it difficult to discern where one arm ends and another starts, and only knowledge of an exact banding pattern for each telomeric region, as it is shown on our map (Fig. 3) can help to distinguish the start/end of a chromosome. Nevertheless, it is recognized here that some telomeres shown on our map could alternatively be the weak points of the chromosome subject to consistent breakage. However, an effort was made to not over-represent the same part of the chromosome on the map. Once the nucleotide sequences of the telomeres of this species are revealed, it will be possible to confirm the location of telomeres using FISH.

### Chromosomes and major landmark description

This description is based on non-stained polytene chromosomes of the salivary glands of IV instar larvae using phase-contrast microscopy. Chromosome 1 is the shortest of the 3 pairs. The pericentromeric region of chromosome 1 forms a large puffy area in regions 11C-12A (Fig. 3). The smaller area of pericentromeric region 11C belongs to the 1R arm, which ends with two faint bands. The bigger part of the pericentromeric region 12A–12B of chromosome 1 ends with a thick and dark band belongs to the 1L arm. The main landmark for the 1R arm is a nucleolar organizer region (NOR) which carries the nucleolus. NOR is situated in region 10C within one division of the centromere. The nucleolus attached to the NOR looks like a large, rounded, uniformly-colored body. The size of the nucleolus is usually several times larger than pericentromeric heterochromatin. Figure 2a represents the nucleolus with usual proportions, although its size varies among individual nuclei. The telomeric region of 1R consists of two small subtelomeric puffs in subdivisions 1A-B. An additional landmark for arm 1L is a thick dark band in the beginning of subdivision 13A. The telomeric region of this arm is often asynaptic in division 19 and may end as either two homologs lying separately from one another, or mostly asynaptic homologs joined back together at the very tip of the telomere, in region 19D as shown on the map (Fig. 3).

Chromosome 2 typically carries the smallest pericentromeric area among all chromosomes. It forms two rounded structures of equal width but with distinct banding patterns in subdivisions 35BC–36A (Fig. 3). The 2R pericentromeric area is puffy in subdivisions 35BC and has a uniform light color with one strong dark band starting subdivision 35C. Sometimes homologs are asynaptic in this region. The 2L pericentromeric area has three thin bands, two of which are more pronounced in subdivision 36A. A big puff starting with the dark band in subdivision 33A, and two dark bands in 35A can be considered as additional landmarks for the 2R arm. A typical pre-telomeric region of the 2R arm has three puffs in subdivisions 21A-C, and the telomeric region ends with the puff and a series of dark heterochromatic bands in subdivision 20A, which are usually underpolytenized. A major landmark for arm 2L is a puff and two dark bands which often fused together and appear as one very dark thick band in subdivision 41B. As an additional landmark the region 38C can be considered, it looks like two thick bands and a puff. The next landmark is located near the telomere: several dark bands usually fused together in region 45C, and the bell-shaped puff in 46A–46B. The 2L arm ends with lighter looking chromatin and a puff in region 48C.

The pericentromeric region of chromosome 3 is usually the largest. However, the arms of the chromosome 3 often cannot be distinguished solely by the morphology of the pericentromeric region, because the banding pattern seems to fluctuate in this area. We consider the main landmark for 3R to be the series of puffs in subdivisions 53B–54A with a particularly large puff in region 53B. An additional landmark is a puffed region in between two dark bands in subdivisions 51B–51C. The two homologs in the telomeric region of the 3R arm are often asynaptic and form a series of bands in region 49A-B. Arm 3L has a series of landmarks: three small puffs in 64C–65A, several thin bands usually fused together, forming a dark and thick looking band in 65C; an additional landmark for 3L is a light area of 3 large puffs in region 68C–69A with a strong band in the beginning of 69B. The telomeric region often looks grainy and diffused in region 70C.

Major landmarks were given nicknames for the ease of reference and placed on the map (see Additional file 1). Photographs of chromosomes with labeled landmarks and nicknames can be found in additional files (see Additional files 2, 3, 4, 5).

### Inversions in JHB

**Fig. 4:**
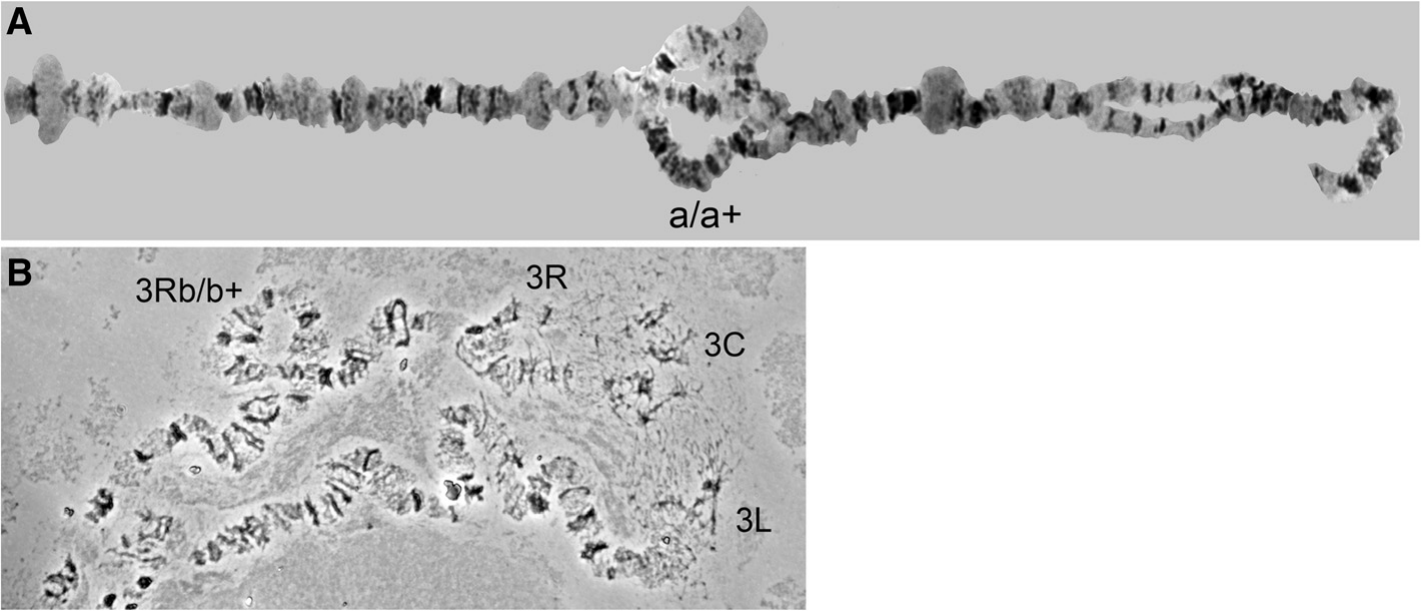
Inversions in *Culex quinquefasciatus.* **a**. Heterozygote for inversion a, tentatively mapped to the arm 2L (a/a+). Polytene chromosomes were obtained from malpighian tubules, and banding pattern does not completely correspond to the cytogenetic map (Fig. 3) based on chromosomes from salivary glands. **b**. Heterozygote for inversion b on the arm 3R (b/b+), near centromere. Centromere is abbreviated as C, right arm as R, left arms as L

During our study we found two distinct inversions in the JHB strain. Both of them are paracentric, which is in agreement with previous reports [8, 14]. One of the inversions was tentatively placed on 2L subdivisions 42A–43C (Fig. 4a), and another is mapped to 3R subdivisions 58C–59C, see Fig. 4b. In additional files this inversion is shown in more detail, also landmarks are placed on the image, to justify the mapping (see Additional file 6). Since this study was not focused on identification of inversions, obtaining more data is required to confirm the boundaries of the inversions. McAbee with coauthors [8] reported the additional inversion belonging to the same chromosome but to the other arm. It is possible that one of the inversions was eliminated from the strain because of inbreeding.

Additionally, there were a few other places on the chromosomes where inversion-like loops were observed, but so far it was impossible to distinguish if those were persistent ectopic contacts together with chromatid asynapsis or inversion heterozygote loops. Nevertheless, it is important to point those places out using the map coordinates for future studies. Their approximate positions on the map (Fig. 3) are: 1L division 14; 2L 40C–39B; 2L40A–39A; 2R 27C–29C; 3R 59C–55C, and 3R 59C–60B.

### The development of the physical genome mapping for *C. quinquefasciatus*

**Fig. 5:**
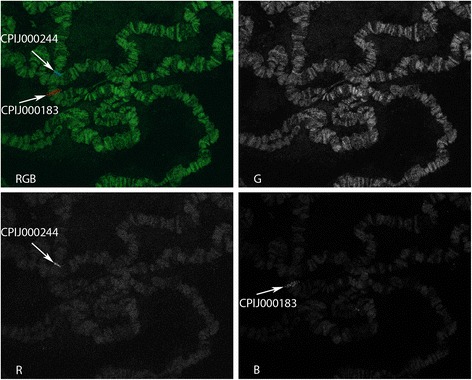
Example of FISH for supercontig 3.3. RGB is a view with Red, Green and Blue channels merged; R – Red channel; G – Green channel, B – Blue channel. The red signal corresponds to the gene CPIJ000183, and the blue signal to CPIJ000244. The results of FISH together with knowledge of the base pair location of each exon within genomic supercontig (see Table 1), allowed inferring directionality of the supercontig within the chromosome as “forward” (see Fig. 3 to find supercontig 3.3 on the map). Refer to Fig. 2 to distinguish telomeres, as the same nucleus was used in both Fig. 2 and Fig. 5

We applied the cytogenetic map developed by this study for the physical mapping of 16 genomic supercontigs, twelve of which are the largest supercontigs of the CpipJ1 genome assembly stored at Vectorbase. Using FISH, the 25 fluorescently labeled DNA fragments were mapped to the *C. quinqefasciatus* chromosomes. These DNA fragments included six genetic markers as cDNA, four of which have correspondence to four genomic supercontigs, and the other two did not correspond to any genomic supercontig; and 19 unique exon sequences of certain genes located in the 12 genomic supercontigs (Table 1, Fig. 3). In order to orient the 12 larger genomic supercontigs, two unique probes were designed to target the flanks of each of the supercontig. The probes were amplified from genomic DNA, labeled with distinct fluorescent dyes and hybridized to polytene chromosomes, as shown in Fig. 5. The location of each hybridization signal was placed on the cytogenetic map (Fig. 3). One of the examples of physical mapping is shown in the additional files (see Additional file 7, for one of the genes from Supercontig 3.1). This way it was possible to determine location of 16 supercontigs and 2 cDNA. We established that five of the supercontigs 3.1, 3.2, 3.3, 3.13, 3.15 have a forward orientation relative to the chromosome numbering, and one supercontig 3.10 is in reverse orientation. The position of probes corresponding to the supercontig 3.8 revealed a putative supercontig misassembly, because one flank of the supercontig is mapped to 2R, and another flank to 2L as shown on Fig. 3. Some probes from supercontigs 3.5, 3.4 and 3.7 produced ambiguous results of FISH, when one probe gave two signals corresponding to different chromosome arms. Thus these supercontigs were assigned to the position of the brightest signal on the chromosomes. Finally, three supercontigs 3.7, 3.9 and 3.12 are missing the information about their direction within the chromosome arm, due to the repetitive nature of one of the two chosen probes. This physical mapping revealed both the correspondence of the new cytogenetic and existing genetic linkage [6] maps as well as the inferred chromosomal location of 32,127,255 base pairs of the assembly with 1333 annotated genes, covering 5.55 % of the genome. In total, 12 matches to the genetic linkage groups were established. Also, 12 matches were made to the mitotic chromosomes physical map described recently [7]. Locations of 13 supercontigs and 2cDNA probes were previously unknown for polytene chromosomes, but were found either for genetic linkage groups [6] or mitotic chromosomes [7], which enabled the integration of the data. Locations of four supercontigs 3.7, 3.8, 3.9, 3.28, as well as orientation for supercontigs 3.13, 3.10, 3.15, 3.3, 3.2 and 3.1 were unveiled for the first time ever in this report. Based on the integration of the new and previously reported data, this constitutes the first step in the production of a golden path for *C. quinquefasciatus*, and will help to improve the current supercontig assembly to the chromosome-based level, which will be reflected in Vectorbase [18].

## Discussion

### Chromosomal naming and physical mapping

In this study we developed an improved cytogenetic map for the polytene chromosomes from the salivary glands of *C. quinquefasciatus* (Fig. 3). Compared with previous photographic maps [8, 15, 16] our map has several new components: 1) images of flattened chromosomes with higher image resolution were utilized to produce a map with detailed banding patterns on the chromosomes; 2) landmarks for each chromosomal arm were established to enable chromosome arm distinction; 3) approximate boundaries for inversion breakpoints were determined (Figs. 3, 4a, 4b); 4) chromosomal arms were completely straightened to facilitate physical mapping. Our map was refined by dividing the chromosomes using 70 numbered divisions and subdivisions lettered from A to D. The total number of divisions was used in concordance with the first drawn map of *C. pipiens* [9]. However, in order to standardize the chromosome nomenclature for genome mapping the chromosomes were numbered in correspondence with the genetic linkage map [6], based on the results of the physical mapping (Fig. 3 and Table 1).

This study clearly indicated the utility of our cytogenetic map for the physical mapping application. In total, 16 supercontigs were physically mapped using FISH. Eleven of them had corresponding coordinates within the genetic linkage map, which enabled the creation of an integrated map. Two more matches were established using cDNA (Table 1, Fig. 3). There were no conflicts found between the order of linkage groups [6], order of supercontigs on mitotic chromosomes [7] and physical mapping order of the supercontigs for polytene chromosomes (Fig. 3). Supercontig 3.8 indicated putative misassembly, when probes from the same supercontig hybridized to the different chromosome arms. Two supercontigs 3.5 and 3.7 have ambiguous results, when one probe gave two signals on different chromosomes. This result for supercontig 3.5 agrees with the data of Hickner *et.al.* [6], which also mapped supercontig 3.5 to chromosomes 2 and 3. These double signals could reflect putative misassembly, gene duplication, but also it could mean that the part of the exon used as a probe could have a similar domain to a sequence on another chromosome, since naturally, during labeling reaction, the probe is being fractured. In the future, it may be possible to find other potential markers which will help clarify the direction of these supercontigs within the chromosome. Thus, our study demonstrated that physical mapping of the *C. quinquefasciatus* genome will help to improve the genome assembly by ordering supercontings, orienting them directionally and suggesting putative misassemblies (Table 1).

### Unique features of *C. quinquefasciatus* polytene chromosomes

Although our study improved the methodology of polytene cytogenetics for *C. quinquefasciatus*, obtaining polytene chromosomes from this species continues to be a challenging and highly time-consuming task. Usually, the lengthy chromosomes, telomere connections and ectopic contacts were observed during slide-preparation and as a result the chromosomes were reluctant to spread on the glass slide. It has been demonstrated on *Drosophila melanogaster* that the positions of ectopic contacts in chromosomes correlate with the location of gene-poor and repeat-rich regions of intercalary heterochromatin which replicate late in S phase of cell cycle [25, 26]. It is not known, weather ectopic contacts are formed due to the under-replication of DNA with the production of sticky ends, or due to the mitotic ectopic recombination in the regions of sequence similarity. Telomeric connections suggest an abundance of repetitive DNA such as transposable elements or satellites in the telomeres of *C. quinquefasciatus* chromosomes, which also may facilitate the formation of multiple ectopic contacts [25, 27], seen in Figs. 1 and 2. In mosquitoes, the abundance of ectopic contacts between chromosomes correlates with the amount of repetitive DNA in their genomes. The amounts of transposable elements are equal to 16 %, 29 % and 47 % for the genomes of *Anopheles gambiae* [28], *C. quinquefasciatus* [5] and *Aedes aegypti* [29] respectively. Almost absent in species from the genus *Anopheles*, ectopic contacts become a significant problem for spreading the chromosomes in *Culex* [8, 9, 16] which possess more transposable elements. Moreover, polytene chromosomes of *Aedes aegypti* become absolutely unspreadable and useless for a cytogenetic analysis or physical mapping due to the ectopic contacts and length [30, 31]. Thus, by knowing the percent of repetitive DNA in the species it could be possible to predict the quality of spreading of polytene chromosomes for the purpose of cytogenetic studies. The threshold is 29 % of the DNA belonging to transposons, above which point the chromosomes would most likely be very difficult to spread due to the ectopic contacts. And vice-versa, it would be expected of the species in which polytene chromosomes make ectopic contacts to possess higher than 16 % of repetitive DNA in their genome. Additionally, *Culex* and *Aedes* genomes are shown to exhibit short interspersion patterns, while *Anopheles* genome has long interspersion genomic patterns [32]. Hence knowledge of the types of transposable elements and their chromosomal locations could also bring insights about the likely quality of chromosomal preparations.

### Improving quality of chromosomal preparations

After four generations of inbreeding, polytene chromosome quality was improved. By using inbreeding we reduced the frequency of chromosomal inversions and improved chromosomal spreading. Also, chromatid homogeneity was noted and fewer instances of homolog asynapsis occurred. This could be explained by the fact that the places of asynapsis are often predictable, and probably include locations of micro indels. By each generation of inbreeding some indels would become absent, and less asynapsis would occur. Interestingly, after the seventh generation of the inbreeding (checked further until fourteenth), the chromosomal quality was not improved any further. Nevertheless, we propose that in insects, inbreeding could be the key-factor for improving polytene chromosome preparation quality, together with food rich in yeast and low larval density. Finally, aceto-orcein staining of non-fixed chromosomes also notably helps with chromosome spreading, although it is useless for *in situ* hybridization technique.

### Application of the new standard cytogenetic map in the future

Inbreeding helped to remove the heterozygosity from the JHB strain and made the chromosomal banding pattern more uniform. While examining the salivary gland polytene chromosomes of *C. quinquefasciatus* Boane strain from Mozambique (unpublished data), we were able to distinguish those chromosomes using the major landmarks described in this manuscript for the JHB strain. Hence, our map will be useful in application to the chromosomes of *C. quinquefasciatus* as a species, and not solely for the JHB strain. However, inversion polymorphism for *C. quinquefasciatus* and banding pattern of the chromosomes of sibling species will have to be determined in future studies.

Special features of *C. quinquefasciatus* polytene chromosomes such as telomere fusions, ectopic contacts and extended length of chromosomal arms prevent a high yield of “readable” slides. So far, polytene chromosome preparation is not efficient on a large scale, which hinders whole-genome physical mapping using polytene chromosomes. Possibly, using mitotic chromosomes as a more easily and more reliably obtainable material could be a good complement to physical mapping on polytene chromosomes as it was demonstrated for the *Aedes aegypti* genome [31, 21] and *C. quinquefasciatus* genome [7]. Since the resolution of physical mapping using mitotic chromosomes is approximately 10 times lower than that of polytene chromosomes, the mitotic chromosomes could be used as a first step of the physical mapping. Then in turn, polytene chromosomes can be utilized for the more detailed fine-scale mapping. Finally, these results can be combined and lead the complete or partial genome reassembly with correction of misassemblies and connection of gaps. In the future, this iterative approach will lead to the construction of a high resolution physical map for *C. quinquefasciatus* genome and enable the creation of a more complete golden path file for this mosquito, and will be reflected in Vectorbase.

The new cytogenetic map presented in this study will serve as a foundation for several types of research. Any genes of interest such as of medical importance and others can be located using physical mapping and placed on the map. This way a detailed fine-scale physical map can be produced by ordering and orienting more genomic supercontigs, and consequently this data will improve the genome assembly. This map is a basis for identifying polymorphic inversions in natural populations of the *C. quinquefasciatus*. Since chromosomal inversions can be linked to adaptation [33], this type of study can improve an understanding of *C. quinquefasciatus* vectorial capacity. This map can serve as a starting point for studying other species from cryptic species *C. pipiens* complex helping to reveal the systematic differences. Finally, all previously mentioned studies will contribute into development of new strategies for the control of vector-borne diseases.

## Conclusions

In this study we developed a new cytogenetic map, with consequent application to the physical mapping of *Culex quinquefasciatus* genome. In total, the locations of 1333 annotated genes were inferred based on the results of physical mapping of 18 genomic supercontigs and 2 genetic markers. Based on this, the truncated genome assembly will be improved. Next, we have identified that JHB strain has at least two inversions. Finally, as a result of this study the naming convention of polytene chromosomes was standardized and linked to that of genetic linkage map of [6]. The new cytogenetic map presented in this study will serve as a foundation for several types of research. It will be used in the development of detailed fine-scale physical map by ordering and orienting more genomic supercontigs, and consequently this data will improve the genome assembly with the goal of placement of most of the supercontigs to the chromosomes. Any genes of interest such as of medical importance and others can be located using this map as a reference. This map is a basis for identifying polymorphic inversions in natural populations of the *C. quinquefasciatus*. Since chromosomal inversions can be linked to adaptation [33], this type of study can improve an understanding of *C. quinquefasciatus* vectorial capacity. This map can serve as a reference for studying other species from *C. pipiens* complex. Finally, all previously mentioned studies will contribute into the development of new strategies for the control of vector-borne diseases.

## Additional files

Additional file 1:
**Cytogenetic map of C. quinquefasciatus chromosomes with labeled and named landmarks.**
Additional file 2:
**Labeled landmarks of chromosomes 2 and 3.**
Additional file 3:
**Labeled centromeres of chromosomes 1 2 and 3, and major landmarks of chromosome 2.**
Additional file 4:
**Major landmarks for chromosome 3.**
Additional file 5:
**Major landmarks for chromosomes 2 and 3.**
Additional file 6:
**Proof of mapping the 3Rb inversion.**
Additional file 7:
**Example of physical mapping of the CPI000004 gene from supercontig 3.1.**

## Abbreviations

*C.*: *Culex*
JHB: Johannesburg
FISH: Fluorescent *in situ* hybridization
PCR: Polymerase Chain Reaction
IDs: Imaginal Discs
GB: Gene Bank
