# Supplementary figures and images for: A standard cytogenetic map of Culex quinquefasciatus polytene chromosomes in application for fine-scale physical mapping
Source: Parasit Vectors. 2015 Jun 6;8:307. doi: 10.1186/s13071-015-0912-4 (PMC4465148; doi:10.1186/s13071-015-0912-4)

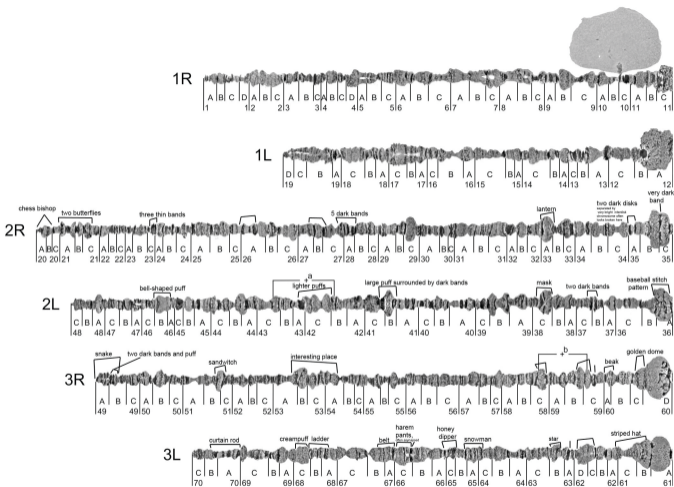

Supplement: Additional file 1: — Cytogenetic map of C. quinquefasciatus chromosomes with labeled and named landmarks. [file 13071_2015_912_MOESM1_ESM.pdf]

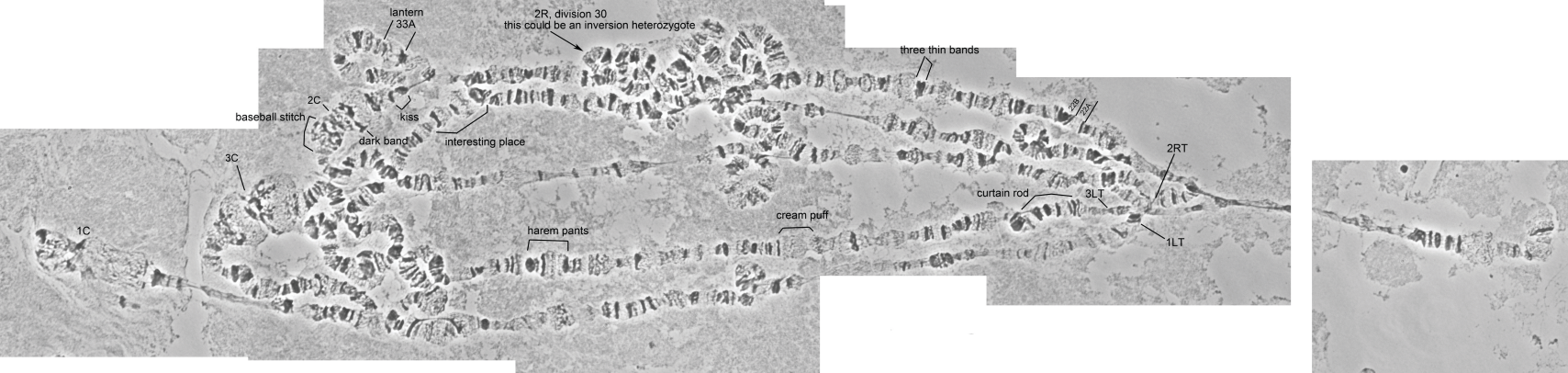

Supplement: Additional file 2: — Labeled landmarks of chromosomes 2 and 3. [file 13071_2015_912_MOESM2_ESM.pdf]

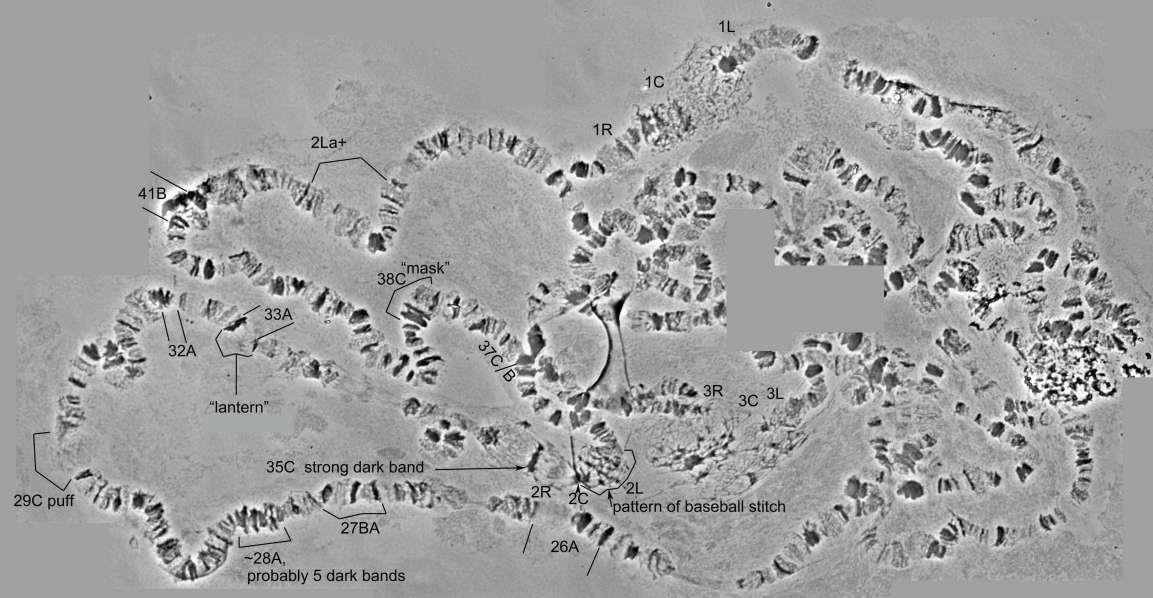

Supplement: Additional file 3: — Labeled centromeres of chromosomes 1 2 and 3, and major landmarks of chromosome 2. [file 13071_2015_912_MOESM3_ESM.pdf]

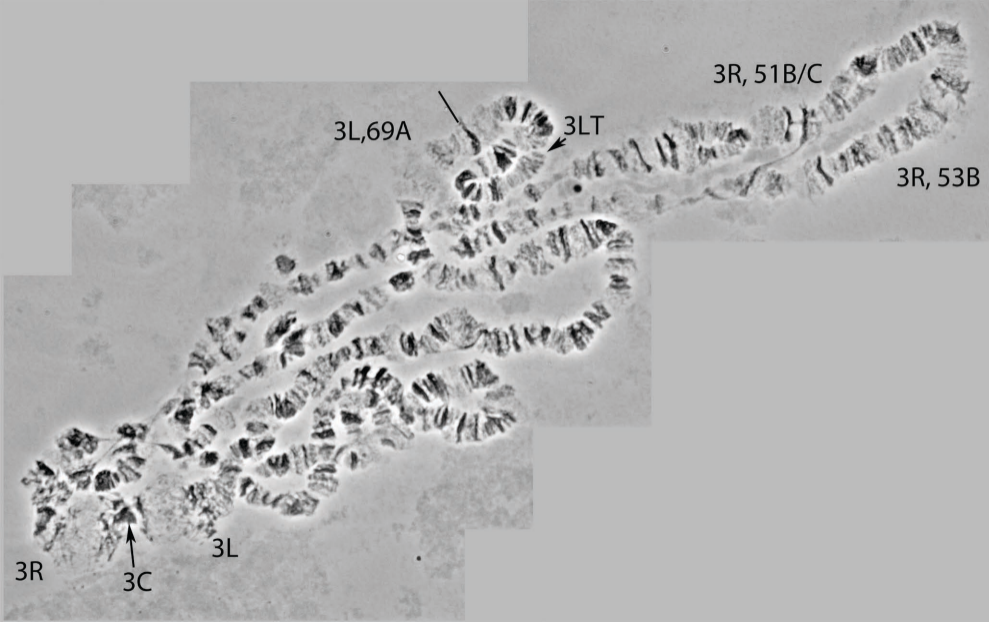

Supplement: Additional file 4: — Major landmarks for chromosome 3. [file 13071_2015_912_MOESM4_ESM.pdf]

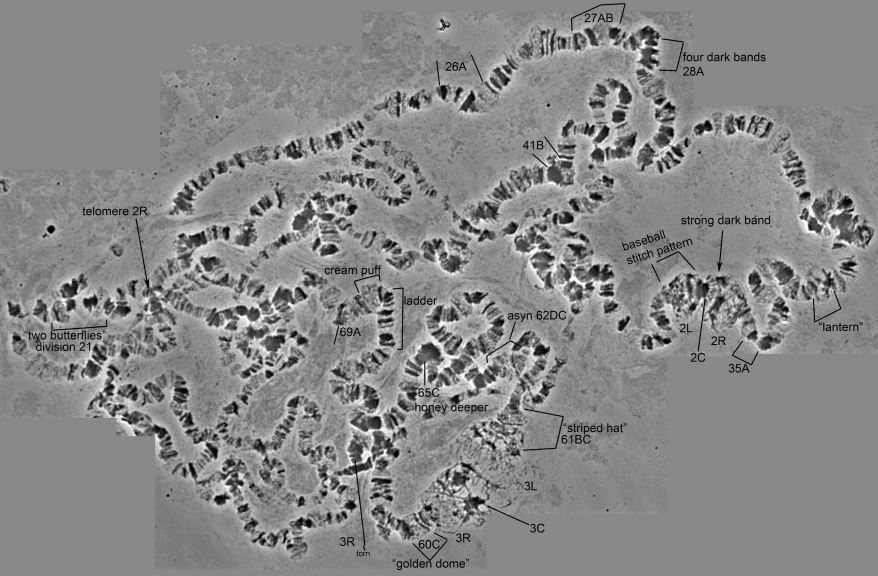

Supplement: Additional file 5: — Major landmarks for chromosomes 2 and 3. [file 13071_2015_912_MOESM5_ESM.pdf]
