# Supplementary material for: A standard cytogenetic map of Culex quinquefasciatus polytene chromosomes in application for fine-scale physical mapping
Source: Parasit Vectors. 2015 Jun 6;8:307. doi: 10.1186/s13071-015-0912-4 (PMC4465148; doi:10.1186/s13071-015-0912-4)
Supplement: Additional file 6: — Proof of mapping the 3Rb inversion. [file 13071_2015_912_MOESM6_ESM.pdf]

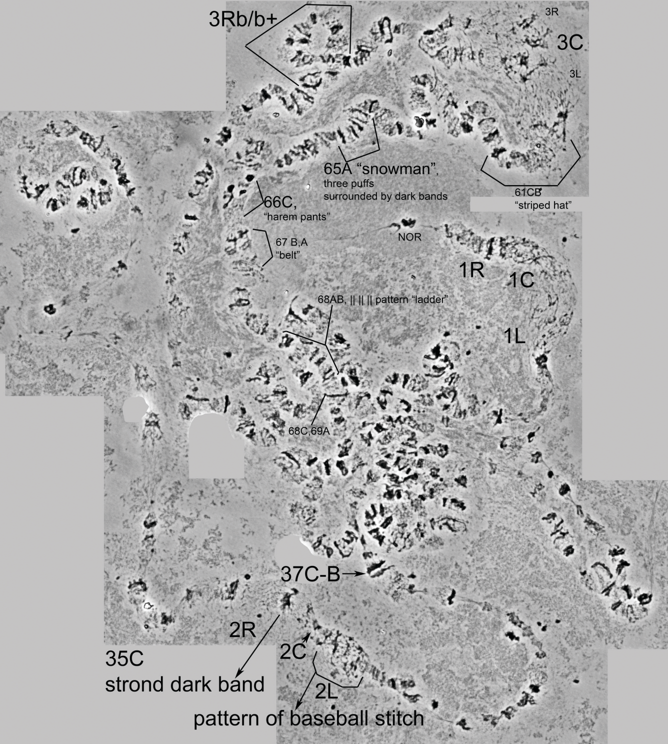
3Rb/b+
3R
3C
3L
65A "snowman",
three puffs
surrounded by dark bands
61CB
"striped hat"
66C,
"harem pants"
67 B,A
"belt"
NOR
1R
1C
1L
68AB, || || || pattern "ladder"
68C,69A
37C-B
2R
2C
2L
35C
strond dark band
pattern of baseball stitch
