# Supplementary material for: A standard cytogenetic map of Culex quinquefasciatus polytene chromosomes in application for fine-scale physical mapping
Source: Parasit Vectors. 2015 Jun 6;8:307. doi: 10.1186/s13071-015-0912-4 (PMC4465148; doi:10.1186/s13071-015-0912-4)
Supplement: Additional file 7: — Example of physical mapping of the CPI000004 gene from supercontig 3.1. [file 13071_2015_912_MOESM7_ESM.pdf]

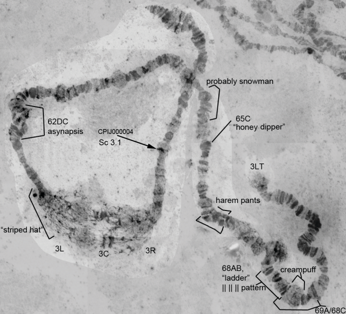
probably snowman
62DC
asynapsis
65C
"honey dipper"
CPIJ000004
Sc 3.1
3LT
harem pants
"striped hat"
3L
3C
3R
creampuff
68AB,
"ladder"
|| || || pattern
69A/68C
